# Association of Helicobacter pylori Infection With the Severity of Psoriasis

**DOI:** 10.7759/cureus.116710

**Published:** 2026-09-22

**Authors:** Marufa Shahrin, Mohammod Abu Hena Chowdhury, Tanzina Begum Chowdhury, Md. Abir Hassan, Arjun Saha, Towfiq Ahmad, Pranam Swapan Dash

**Affiliations:** 1 Department of Dermatology, Shaheed Suhrawardy Medical College, Dhaka, BGD; 2 Department of Dermatology, Bangladesh Medical University, Dhaka, BGD; 3 Department of Dermatology, Sarkari Karmachari Hospital, Dhaka, BGD; 4 Department of Dermatology, Sir Salimullah Medical College, Dhaka, BGD; 5 Department of Dermatology, Directorate General of Health Services, Dhaka, BGD; 6 Department of Dermatology, Rajshahi Medical College Hospital, Rajshahi, BGD

**Keywords:** disease severity, helicobacter pylori, psoriasis, psoriasis area and severity index (pasi), stool antigen test

## Abstract

Background: Psoriasis is an inflammatory, immune-mediated disease with a multifactorial etiopathogenesis and a wide spectrum of clinical presentation. It has been proposed that *Helicobacter pylor*i (*H. pylori*) infection acts as a possible etiological factor for psoriasis.

Methods: Twenty-nine patients diagnosed with psoriasis were included in Group A, and an equal number of non-psoriatic individuals were included in Group B based on predefined inclusion and exclusion criteria. Psoriasis severity was assessed using the Psoriasis Area and Severity Index (PASI) score.

Results: The epidemiological profile showed no significant differences in age or sex between patients and healthy individuals (age p=0.480; sex p=0.597). Among psoriasis patients, 23 (79.3%) had the disease for less than 10 years, and the mean disease duration was 4.76 ± 4.78 years (range: one to 20 years). A significant association was found between *H. pylori *infection and psoriasis, with nine (31.0%) of psoriasis patients testing positive for *H. pylori* stool antigen compared to only two (6.9%) in controls (p=0.019).

Conclusion: *H. pylori* infection has a significant association with the severity of psoriasis. This finding highlights a probable role of bacterial infection in exacerbating immune-mediated skin conditions. Therefore, it can be proposed that psoriasis patients with high PASI scores may be screened for *H. pylori* infection by a fecal antigen test.

## Introduction

Psoriasis is a common, immune-mediated, recurrent disease of skin and joints with a wide spectrum of clinical presentation. A systematic worldwide review found that adult psoriasis prevalence varied from 0.51% to 11.43% [1]. In Bangladesh, the point prevalence of all types of psoriasis is 0.7% [2]. While the prevalence of *Helicobacter pylori* (*H. pylori*) infection is 92% among the asymptomatic population in Bangladesh [3].

There are many types of psoriasis, such as plaque psoriasis, palmoplantar psoriasis, guttate psoriasis, pustular psoriasis, inverse psoriasis, and erythrodermic psoriasis. Among these, plaque psoriasis is the most common. Typically, the lesions are symmetrically distributed, with a preference for extensor surfaces of the limbs, scalp, and nails [4].

The pathogenesis of psoriasis is complicated. It’s a complex interaction between the immune system, genetics, and environmental factors. It has been demonstrated that Th17 cells play a key role in the pathogenesis of psoriasis. Numerous immune cells and cytokines, including tumor necrosis factors, interleukin (IL)-17, IL-22, and IL-23, are involved in the pathogenesis of psoriasis [5].

Environmental variables have been identified and acknowledged as having significant involvement in psoriasis onset and progression. Psoriasis is triggered by environmental factors like stress, surgery, alcoholism, smoking, and infection. Numerous microorganisms are responsible for it; mostly bacteria, fungi, and viruses contribute to the pathophysiology of psoriasis. The clinical correlation between guttate psoriasis and streptococcal pharyngitis is well established, indicating a bridge between psoriasis and bacterial infection [6].

It is believed that *H. pylori *is one of the bacteria capable of triggering psoriasis [7]. *H. pylori *is a gram-negative bacterium. It usually inhabits the gastric mucosa and can cause peptic ulcer, gastritis, and gastric cancer [8]. This infection has been proposed as a culprit of various extraintestinal gastrointestinal disorders. Several studies indicate that *H. pylori* infections play roles in the pathogenesis of various skin diseases [9], notably in idiopathic chronic urticaria [10].

Through the gut-skin axis, it has recently been shown that the gut microbiome may be a significant factor in psoriasis [11]. Some studies reported that *H. pylori* can trigger not only local inflammatory responses but also systemic inflammatory reactions through the uncontrolled release of interleukins [9].

*H. pylori* infection has an effect on psoriasis patients. The severity of psoriasis was considerably higher in *H. pylori*-positive psoriasis patients than in *H. pylori*-negative psoriasis patients [12].

However, a few studies contrast the results of the above-stated studies and show no association between psoriasis and *H. pylori* infection. Still, the relationship between *H. pylori* infection and psoriasis is an intriguing and challenging field of research.

## Materials and methods

This was a cross-sectional comparative study. The study was conducted in the Department of Dermatology and Venereology, Bangladesh Medical University (BMU), Dhaka, Bangladesh, from May 2023 to March 2025. The number of subjects in Group A was 29, and in Group B it was 29; the total sample size was 58.

Study population

Group A included individuals with diagnosed psoriasis, and Group B included non-psoriatic individuals. Participants were recruited from both the outpatient and inpatient Departments of Dermatology and Venereology at BMU. A consecutive sampling technique was used for participant selection.

Data collection technique

The dermatological and physical examination was performed by the principal investigator of the study, and the diagnosis of psoriasis was further confirmed by the supervisor of this study. Informed written consent was obtained from each subject when he/she wished to be a sampling unit of the study after explaining the utility of this study. All participants were interviewed using a semi-structured questionnaire (Appendix A). A thorough history, including age, gender, duration of psoriasis, and concomitant medications, was taken, and a relevant physical examination was done. The Psoriasis Area and Severity Index (PASI) was calculated for each patient [13]. Then, patients with psoriasis underwent the required laboratory investigation, which was stool antigen for *H. pylori*. *H. pylori *detection in stool samples was performed using the CerTest *H. pylori* stool antigen test (Certest Biotec, Zaragoza, Spain), following the manufacturer’s instructions.

Data collection tools

A checklist was prepared for each patient, including the PASI score measuring tool, tools for physical examination, and laboratory investigation reports. Written informed consent was obtained from each participant in either English or Bangla. A semi-structured questionnaire was developed for data collection. Photographs of the study subjects were taken after obtaining informed consent.

Data processing and analysis

Quantitative variables were expressed as mean ± standard deviation (SD), while qualitative variables were summarized using frequencies and percentages. Chi-square (χ²) test (for categorical variables), Mann-Whitney U test (for non-parametric two-group comparisons) and Kruskal-Wallis test (for non-parametric three-group comparisons) were used. Statistical analysis was conducted using IBM SPSS software version 26.0 (IBM Corp., Armonk, NY, USA). A p-value <0.05 was considered statistically significant. Tables were used to present the study results. At the end of the study, a summary and conclusion were provided to synthesize the findings.

Ethical consideration

The study was approved by the Institutional Review Board (IRB) of BMU (Approval No: BSMMU/2023/14985). The eligibility of the participants was assessed, and they were informed about the procedure and the study objectives, and were told that there was no chance of any harm to the individuals by inclusion in the study. Individuals were also informed that they were free to refuse to participate or to withdraw at any time. Every participant willingly agreed to give their stool as a sample. 

Quality assurance

The study was conducted in the Department of Dermatology under the guidance of dermatologists and personnel experienced in the field. Regular monitoring and outcome assessments were performed to ensure the quality of data collection. Initially, four to five samples were enrolled for training and piloting before proceeding with the main study. Stool samples were collected under the direct supervision of the study supervisor. All collected data were carefully verified and cross-checked to maintain accuracy and reliability.

## Results

A total of 58 participants were included in the study, comprising 29 patients with psoriasis (Group A) and 29 non-psoriatic controls (Group B). The mean age was 31.3 ± 15.9 years in the psoriasis group and 34.2 ± 15.4 years in the control group. The median ages were 30 and 32 years, respectively, with ranges of four to 70 and six to 70 years. The distribution of age categories did not differ significantly between the two groups (χ²=5.292, df=6, p=0.507). Similarly, sex distribution was comparable, with males comprising 14 (48.3%) participants in the psoriasis group and 12 (41.4%) in the control group, while females comprised 15 (51.7%) and 17 (58.6%), respectively (χ²=0.279, df=1, p=0.597). Among the psoriasis patients, 19 (65.5%) had a disease duration of < 5 years, and 10 (34.5%) had a duration of ≥5 years. The mean disease duration was 4.76 ± 4.78 years (Table 1).

**Table 1:** Characteristics of the study group (n=58). Values are presented as mean ± SD or n (%). Age-group and sex comparisons used the chi-square (χ²) test. Disease duration was assessed only in Group A. P < 0.05 was considered statistically significant.

Characteristics	Group A (n=29)	Group B (n=29)	Test statistic	df	p-value
<18 years	7 (24.1%)	3 (10.3%)	χ² = 5.292	6	0.507
18-20 years	0 (0.0%)	2 (6.9%)			
21-30 years	9 (31.0%)	9 (31.0%)			
31-40 years	5 (17.2%)	8 (27.6%)			
41-50 years	4 (13.8%)	2 (6.9%)			
51-60	3 (10.3%)	3 (10.3%)			
>60 years	1 (3.4%)	2 (6.9%)			
Mean ± SD (years)	31.3 ± 15.9	34.2 ± 15.4			
Median (years)	30	32			
Range (years)	4–70	6–70			
Male	14 (48.3%)	12 (41.4%)	χ² = 0.279	1	0.597
Female	15 (51.7%)	17 (58.6%)			
Disease duration <5 years	19 (65.5%)	N/A			
Disease duration >5 years	7 (24.1%)	N/A			
Mean ± SD duration (years)	4.76 ± 4.78	N/A			
Range (years)	1–20	N/A			

Among psoriasis patients, PASI scores differed significantly according to *H. pylori* status. The mean PASI score was 21.69 ± 5.14 among *H. pylori*-positive patients compared with 7.23 ± 7.13 among *H. pylori*-negative patients. The corresponding median PASI scores were 22.2 and 7.05, with ranges of 15.6-30.0 and 0.6-33.1, respectively. The Mann-Whitney U test demonstrated a statistically significant difference in PASI scores (U=171.0, p<0.001; exact two-sided p=0.000147). Thus, psoriasis patients with detectable *H. pylori* stool antigen had substantially higher disease severity scores than those without detectable infection (Table 2, Figure 1).

**Table 2:** Psoriasis Area and Severity Index (PASI) score according to Helicobacter pylori (H. pylori) status (n=58). Values are presented as mean ± SD, median, and range. Mann–Whitney U test was used. Exact two-sided p=0.000147. P < 0.05 was considered statistically significant. Reference: [13]

PASI score	*H. pylori-*positive (n=9)	*H. pylori*-negative (n=20)	U	p-value
Mean ± SD	21.69 ± 5.14	7.23 ± 7.13	U = 171.0	0.000147
Median	22.2	7.05		
Range	15.6–30.0	0.6–33.1		

**Figure 1:**
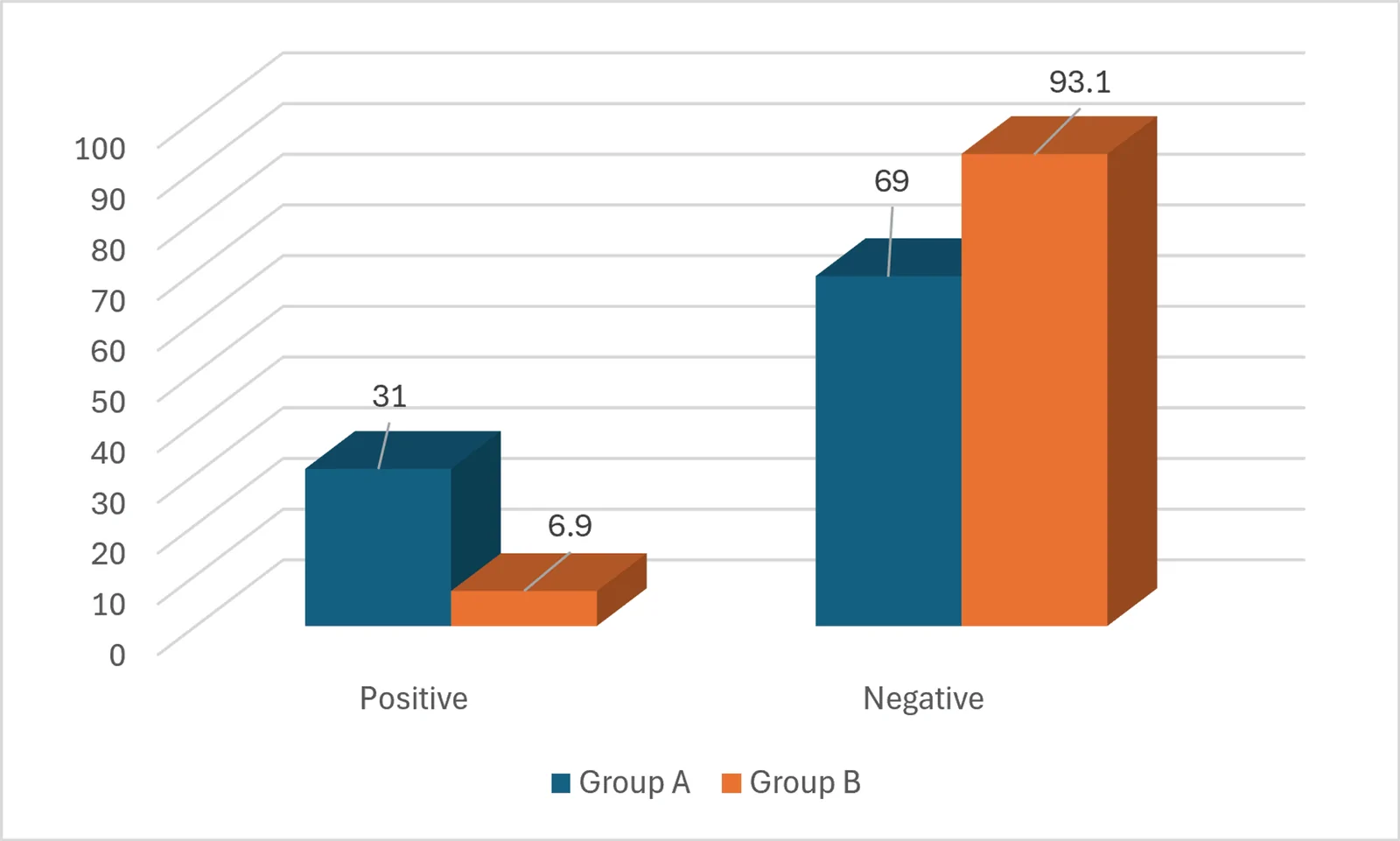
Comparison of Helicobacter pylori stool antigen status between psoriasis patients and healthy controls. The figure presents the proportion of participants with positive and negative *Helicobacter pylori *stool antigen results in patients with psoriasis (Group A, n = 29) and healthy controls (Group B, n = 29). Overall, 58 participants were included in the analysis.

When psoriasis patients were categorized by clinical severity, nine (31.0%) had mild disease, eight (27.6%) had moderate disease, and 12 (41.4%) had severe disease. Mean age was 32.9 ± 18.2 years in the mild group, 26.8 ± 16.6 years in the moderate group, and 33.1 ± 14.5 years in the severe group. Age did not differ significantly across severity categories (Kruskal-Wallis H=0.924, df=2, p=0.630). Sex distribution was also similar across the three groups: males accounted for four (44.4%), four (50.0%), and six (50.0%) participants in the mild, moderate, and severe groups, respectively (χ²=0.077, df=2, p=0.962). The disease-duration category was likewise not significantly associated with severity (χ²=0.721, df=2, p=0.697). Therefore, age, sex, and duration of disease were not significantly different across the psoriasis severity groups (Table 3).

**Table 3:** Participant characteristics by psoriasis severity (n=58). Values are presented as mean ± SD or n (%). Age was compared using the Kruskal–Wallis test; sex and disease-duration category were compared using chi-square (χ²) tests. The supplied raw dataset labels duration categories as <5 and >5 years. P < 0.05 was considered statistically significant.

Variable	Mild (n=9)	Moderate (n=8)	Severe (n=12)	Test statistic	df	p-value
Age (years), mean ± SD	32.9 ± 18.2	26.8 ± 16.6	33.1 ± 14.5	H = 0.924	2	0.630
Age (years), median	24.0	27.5	32.5			
Age (years), range	16–70	4–50	8–56			
Male	4 (44.4%)	4 (50.0%)	6 (50.0%)	χ² = 0.077	2	0.962
Female	5 (55.6%)	4 (50.0%)	6 (50.0%)			
Disease duration <5 years	5 (55.6%)	6 (75.0%)	8 (66.7%)	χ² = 0.721	2	0.697
Disease duration >5 years	4 (44.4%)	2 (25.0%)	4 (33.3%)			

Comparison of demographic and disease-duration characteristics according to *H. pylori *status showed no statistically significant differences. The mean age was 30.4 ± 17.3 years among *H. pylori*-positive patients and 31.7 ± 15.8 years among *H. pylori*-negative patients; the difference was not significant (Mann-Whitney U=93.5, p=0.887). Males comprised five (55.6%) of the *H. pylori*-positive group and nine (45.0%) of the negative group, while females comprised four (44.4%) and 11 (55.0%), respectively (χ²=0.277, df=1, p=0.599). Mean disease duration was 3.44 ± 2.83 years in the positive group and 5.35 ± 5.39 years in the negative group, with no significant difference (Mann-Whitney U=76.0, p=0.504) (Table 4).

**Table 4:** Participant characteristics by Helicobacter pylori (H. pylori) infection (n=58). Values are presented as mean ± SD, median, range, or n (%). Age and disease duration were compared using the Mann–Whitney U test; sex was compared using the chi-square (χ²) test. P < 0.05 was considered statistically significant.

Variable	*H. pylori-*positive (n=9)	*H. pylori-*negative (n=20)	Test statistic	df	p-value
Age (years), mean ± SD	30.4 ± 17.3	31.6 ± 15.8	U = 93.5		0.887
Age (years), median	30.0	27.5			
Age (years), range	4–56	12–70			
Male	5 (55.6%)	9 (45.0%)	χ² = 0.277	1	0.599
Female	4 (44.4%)	11 (55.0%)			
Disease duration (years), mean ± SD	3.44 ± 2.83	5.35 ± 5.39	U = 76.0		0.504
Disease duration (years), median	2	2			
Disease duration (years), range	1–10	1–20			

The relationship between *H. pylori* infection and psoriasis severity was further examined. None of the patients with mild psoriasis were *H. pylori*-positive; all nine (100.0%) were negative. Among patients with moderate psoriasis, two (22.2%) were *H. pylori*-positive and six (30.0%) were negative. In the severe group, seven (77.8%) were *H. pylori*-positive and five (25.0%) were negative. The distribution of *H. pylori* status across severity categories was statistically significant (χ²=8.364, df=2, p=0.015). This pattern showed that *H. pylori* positivity was concentrated predominantly among patients with severe psoriasis (Table 5).

**Table 5:** Psoriasis severity by Helicobacter pylori (H. pylori) infection (n=58). Values are presented as n (%). A chi-square test (χ²) was used. P < 0.05 was considered statistically significant. PASI: Psoriasis Area and Severity Index [13]

Disease severity (PASI score)	*H. pylori-*positive (n=9)	*H. pylori-*negative (n=20)	Test statistic	df	p-value
Mild (<5)	0 (0.0%)	9 (45.0%)	χ² = 8.364	2	0.015
Moderate (5-10)	2 (22.2%)	6 (30.0%)			
Severe (>10)	7 (77.8%)	5 (25.0%)			

## Discussion

This was a cross-sectional comparative study, and it showed *H. pylori* infection as an important risk factor in psoriasis, especially with higher disease severity. Despite the similar demographic characteristics between psoriasis patients and healthy controls, *H. pylori* stool antigen was significantly more prevalent in psoriasis patients compared to controls. In addition, PASI scores were notably higher in *H. pylori *stool antigen-positive patients than those without infection, showing that the infection was an important contributor rather than just a cofactor for the severity of the disease.

The current study demonstrated that *H. pylori* infection was significantly more common among patients with psoriasis than controls. Similar findings were reported in other studies, as there was a positive association between psoriasis and *H. pylori* infection [3,12,14]. In a similar study, a greater number of *H. pylori *was obtained from psoriasis patients, and it was rightly speculated that chronic infection could boost the lingering systemic inflammation caused by the disease [15]. In addition, there was a recent systematic review and meta-analysis that found an association between *H. pylori *infection and psoriasis using moderate heterogeneity among published studies [16].

An important finding of this study was the significant correlation between *H. pylori* infection and disease severity. Stool antigen-positive patients had significantly higher PASI scores than those with negative results, and all infected patients belonged to the severe psoriasis group. Our results are in line with previous studies showing much higher PASI scores among patients who were *H. pylori*-positive [3,14,17]. Histologically proven *H. pylori *infections were also demonstrated to increase with the severity of psoriasis [18]. However, they found that there was no longitudinal association between *H. pylori* infection and psoriasis after an adjustment for potential confounders, suggesting more methodologically sound prospective studies will be needed [19].

There are a number of mechanisms that could potentially account for the observed association. Chronic *H. pylori *infection causes continuous immune activation and induces overproduction of various kinds of inflammatory cytokines such as TNF-α, IL-17, and IL-23, which are main contributors to psoriasis pathogenesis [5,6]. Moreover, *H. pylori *may contribute to molecular mimicry, with sustained immune activation contributing to long-term systemic inflammation [8]. The gut-skin axis has likewise been established as a potential mechanism between the gut microbiota and inflammatory skin diseases. Changes in gut microbiota might affect immune regulation and worsen psoriasis [11].

Our results may have clinical relevance, especially in the context of Bangladesh, where *H. pylori *infection is extremely common in the general population. For selected patients with moderate-to-severe psoriasis, this may be the reason to screen for *H. pylori* infection. However, more prospective studies and randomized clinical trials are needed to confirm whether improvement in psoriasis outcomes occurs with eradication of *H. pylori*.

This study has its strengths: a control group comparable in age and sex, fecal antigen for active detection of *H. pylori* infection, and assessment of the severity of the disease through the validated PASI score. While these results seem very promising, some limitations need to be considered. It was a relatively small, single-center, retrospective cohort study, which adds potential bias and makes generalization of results not universal. In addition to that, the cross-sectional design does not provide evidence for causality, and no inflammatory biomarkers or bacterial virulence factors were investigated.

Overall, the findings of this study support the growing evidence that *H. pylori* infection is associated with increased psoriasis severity [3, 16]. Further multicenter prospective studies with larger sample sizes are warranted to confirm these findings and to evaluate the potential therapeutic benefit of *H. pylori* eradication in patients with psoriasis.

## Conclusions

This study provides evidence of a significant association between *H. pylori *infection and increased severity of psoriasis. The presence of *H. pylori* was more common in psoriasis patients and was linked to more severe disease manifestations. The consistent trend of higher psoriasis severity among *H. pylori*-positive patients suggests a probable role of bacterial infection in exacerbating immune-mediated skin conditions. The mechanisms underlying this association may involve chronic inflammation, immune system modulation, or gut-skin axis interactions.

## Appendices

Appendix A

Data Collection Sheet

1.      Name: ……………………………………………………

2.      Age: ……… years

3.      Gender:     Male: 1          Female: 2

4.      Address: ……………………………………………………………………………………

5.      Mobile no………………………………........

6.      Duration of disease:                               year

7.      Previous history of gastric surgery:

         1) Yes     2) No

 8.      History of any other disease which is associated with *H. pylori*:

         1) Yes     2) No, if yes, disease name:

9.      Personal history of GIT symptoms

         1) Present       2) Absent

10.  Type of psoriasis:

       1) Plaque 2) Guttate 3) Nail 4) Inverse 5) Sebopsoriasis 6) Pustular 7)  Erythrodermic 8) Others

11.  Histopathology of suspected skin lesion:

      1) Psoriasis    2) Others

12.  Drug history: (Drugs taken in last 3 months)

       Systemic corticosteroid therapy

       1) Yes     2) No

       Methotrexate therapy

       1) Yes     2) No

      Cyclosporine therapy

      1) Yes     2) No

      Systemic retinoids

     1) Yes     2) No

      Biologics therapy

      1) Yes     2) No

     Antibiotics for *H.pylori* eradication in last 12 weeks

     1) Yes     2) No

     PPI in last 1 week

     1) Yes     2) No

13. Lab Findings

      Stool antigen for *H.pylori*

14. Assessment of severity of psoriasis by using the PASI score:
